# Ionic Liquid Modified Polymer Gel for Arsenic Speciation

**DOI:** 10.3390/molecules29040898

**Published:** 2024-02-18

**Authors:** Ivanka Dakova, Irina Karadjova

**Affiliations:** Faculty of Chemistry and Pharmacy, University of Sofia “St. Kliment Ohridski”, 1, James. Bourchier Blvd.1, 1164 Sofia, Bulgaria; karadjova@chem.uni-sofia.bg

**Keywords:** ionic liquid-based polymer gel, inorganic and organic arsenic species, arsenic speciation, surface waters

## Abstract

A new ionic liquid modified polymer gel containing methylimidazolium groups (poly(MIA)) is proposed as a sorbent for the separation and enrichment of trace inorganic and organic arsenic species in surface waters. The poly(MIA) was synthesized by chemical modification of polymeric precursor using post-polymerization modification of poly(glycidyl methacrylate-co-trimethylolpropane trimethacrylate). The composition, structure, morphology, and surface properties of the prepared particles were characterized using elemental analysis, Fourier transform infrared spectroscopy, scanning electron microscopy, and nitrogen adsorption–desorption measurements. Optimization experiments showed that at pH 8, monomethylarsonic acid (MMAs), dimethylarsinic acid (DMAs), and As(V) were completely retained on the poly(MIA), while the sorption of As(III) was insignificant. The desorption experiments revealed that due to the weaker binding of organic arsenic species, selective elution with 1 mol/L acetic acid for MMAs + DMAs, followed by elution with 2 mol/L hydrochloric acid for As(V), ensured their quantitative separation. The adsorption kinetic and mechanism were defined. The analytical procedure for As(III), As(V), MMAs, and DMAs determination in surface waters was developed and validated through the analysis of certified reference material.

## 1. Introduction

Arsenic is a chemical element of the earth’s crust and is widely distributed throughout the environment. Arsenic exists in air, soil, biota, and water in the form of more than 40 chemical forms with widely varying mobility and toxicity [1]. Trivalent arsenicals are, as a rule, more toxic than pentavalent arsenicals, and inorganic species are more toxic than the organic ones, but trivalent organic arsenic compounds can be more toxic than trivalent inorganic forms [2,3]. Drinking water (inorganic arsenic might be naturally present in ground waters) used in daily life and contaminated food and beverages are the main sources of toxic inorganic arsenic (As(III) and As(V)) species, leading to adverse chronic health effects [4,5]. On the contrary, high arsenic concentrations in sea food are almost harmless due to the low toxicity of the accumulated organic arsenic species (arsenobetaine, arsenocholine, arsenosugars). This is why the aim of speciation analysis in the case of arsenic may be to ensure the complete determination of all arsenic chemical species existing in the sample (however, not all known arsenic species exists in a given sample), or more practically, to allow for the reliable determination of only toxic arsenic forms, e.g., inorganic arsenic and both organic arsenicals (monomethylarsonic acid (MMAs) and dimethylarsinic acid (DMAs)), which still display a certain degree of carcinogenicity. The application of the chromatographic separation of arsenic forms combined with their detection with a sensitive instrumental method is probably the best analytical solution, but it requires expensive equipment, the procedure is labor-intensive and time-consuming, and it is not particularly suitable for sample throughput analysis [6]. The development of fast and selective methods for the quantification of only toxic forms of arsenic based on solid-phase extraction (non-chromatographic approach) with a suitable sorbent is preferable and an effective approach to control the quality of a large number of samples. The essential conditions for the application of this approach are: relatively easy and reproducible synthesis of the sorbent from accessible reagents; the sorbent should be characterized with high extraction efficiency and selectivity toward the target species; the whole analytical procedure is simple and easy to manipulate. Recently, arsenic speciation based on non-chromatographic approaches using solid-phase extraction has often been used due to the abovementioned simplicity, low cost, robustness, and flexibility [7,8,9,10,11,12,13]. 

Different strategies have been developed for the separation of As species based on SPE and their further quantification:

- Multi-sorbent based SPE procedure: All arsenic species are retained on different sorbents and then selectively eluted using suitable elution agents [14,15,16,17,18,19];
- Retention of all organic and inorganic As species on one sorbent, followed by their simultaneous elution and subsequent chromatographic separation and determination [20,21];
- A method based on ultrasound-assisted dispersive solid–liquid multiple-phase microextraction [22];
- The selective retention of As species is achieved by combining pH changes and pyrrolidine dithiocarbamate as an auxiliary reagent [23].

Ionic liquid-based polymer gels (IL-PGs, also called poly(ionic liquid)s or ionogels) are a subclass of polyelectrolytes with repeating ionic units in their polymer chains [24,25]. They are usually synthesized by the polymerization of an ionic liquid (IL) monomer or by the post-polymerization modification of uncharged polymers with IL [26]. Due to the combination of the unique properties of the IL and the macromolecular architecture, these materials have potential applications in various fields, such as analytical chemistry, biotechnology, gas separation, catalysis, etc. [27]. IL-PGs possess high stability (thermal, chemical, and mechanical) and an abundance of functional groups, which allows them to be used as effective sorbents for SPE [28]. Moreover, by selecting an appropriate type of IL immobilized in the polymer gel, it is possible to achieve high adsorption capacity, extraction efficiency, and selectivity toward different classes of analytes, including chemical species of elements. However, in analytical applications they are usually used in the combination with chelating agents.

In this paper, we report the synthesis and characterization of a new ionic liquid-based polymer gel and its application for arsenic determination and speciation in surface waters. The newly synthesized sorbent under optimal conditions showed high extraction efficiency toward inorganic (As(III) and As(V)) and organic (MMAs and DMAs) arsenic species without using any additional chelating reagent. According to kinetics studies, the adsorption process follows a pseudo-second-order kinetic model. The adsorption equilibrium data fit well with the Langmuir isotherm model. The quantitative separation of retained arsenic species is achieved by selective elution. The developed analytical procedure solves all problems for arsenic speciation:

- Separation of As species without using reagents for reduction or oxidation and any subtraction from total amount;
- Low determination limits achieved due to enrichment factors obtained;
- Low matrix interferences result in practical analytical procedure useful for monitoring campaigns for all types of surface waters.

The proposed analytical procedure for As(III), As(V), DMAs, and MMAs speciation in surface waters was validated through the added–found method and by the analysis of certified reference materials. 

## 2. Results

### 2.1. Synthesis and Characterization of Poly(MIA)

**Figure 1:**
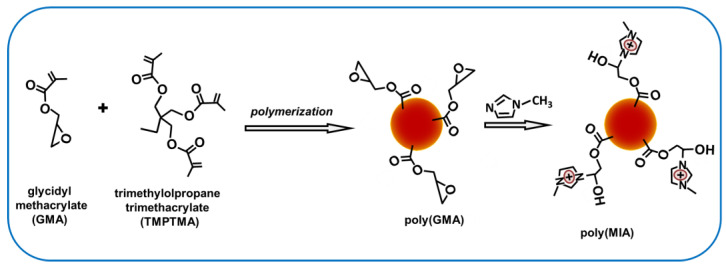
Schematic representation of the poly(MIA) synthesis.

The new poly(MIA) sorbent material was synthesized via the chemical modification of a polymeric precursor (Figure 1). Initially, poly(glycidyl methacrylate-co-trimethylolpropane trimethacrylate) polymer gel (poly(GMA)) was prepared by the precipitation copolymerization of glycidyl methacrylate and trimethylolpropane trimethacrylate in the presence of 2,2′-azo-bis-isobutyronitrile as an initiator and acetonitrile as a porogenic solvent. In the next step, the poly(GMA) particles were modified by chemical binding with 1-methylimidazole (MIA) to obtain IL-PG—poly(MIA). The degree of MIA binding was established by elemental analysis of the synthesized poly(MIA). The results obtained (12.04 wt.% N) revealed that 1-methylimidazole was successfully introduced with a high degree onto the P(GMA) surface. 

The FTIR spectra presented in Figure S1 further confirm the successful interaction between the epoxy groups in poly(GMA) and the MIA, leading to the production of poly(MIA). A typical methacrylate ester band at 1734 cm^−1^ was observed in both spectra. In the spectrum of the poly(GMA), the two peaks found at 908 and 840 cm^−1^ are related to the vibration of the epoxy group. After MIA modification, the intensity of these two peaks significantly decreased. At the same time, a strong and broad band between 3100 and 3700 cm^−1^ appeared in the spectrum of the poly(MIA), which can be attributed to the combination of the stretching vibrational bands of the hydroxyl and secondary amine groups [29]. In addition, the band at 1575 cm^−1^ is typical of the N−H vibration [30]. 

**Figure 2:**
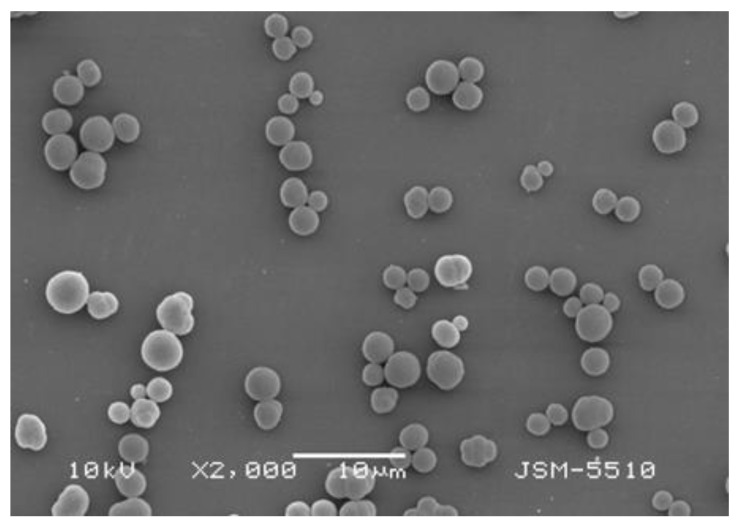
SEM image of poly(MIA).

The morphology and shape of the polymer gel particles was studied by SEM. The mean diameter of the poly(MIA) particles, determined from the micrograph presented in Figure 2, was 2.34 µm. The particles had an almost spherical shape. The specific surface area (*S*_BET_ = 27 m^2^/g), total pore volume (*V*_total_ = 0.10 m^3^/g), and average pore diameter (*D*_average_ = 15 nm) for the poly(MIA) particles were determined from nitrogen adsorption–desorption isotherms. The *D*_average_ value confirms the mesoporous structure of the polymer gel particles.

### 2.2. Extraction Efficiency of Poly(MIA) toward Four Arsenic Species

Several important parameters were optimized in order to assess the applicability of the poly(MIA) for the selective determination of four arsenic species: As(III), As(V), MMAs, and DMAs. 

#### Effect of pH of Sample Media on the Retention of as Species

**Figure 3:**
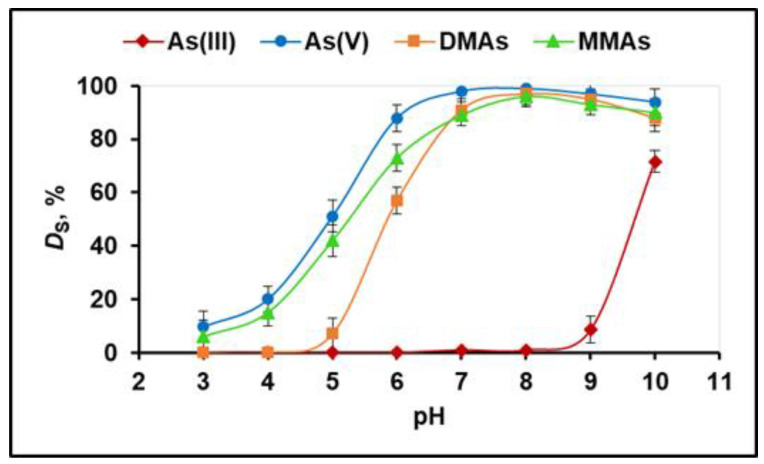
pH dependence of the degree of sorption (*D*_S_, %) of As(III), As(V), DMAs, and MMAs ions with poly(MIA) (three parallel experiments).

The acidity of the solution is a key parameter controlling both the degree of sorption and degree of elution of As(III), As(V), DMAs, and MMAs ions because the pH value of the sample solution influences the degree of ionization of As species. The effect of pH on the adsorption of the four arsenic species was tested separately after the equilibration of 25 mg of poly(MIA) with 10 mL of sample solutions containing 2 µg/mL of the target analytes under different pH values in the range of 3–10. As seen in Figure 3, at pH = 3–8, the degree of sorption of As(III) was less than 1% since As(III) mostly exists in the form of neutral H_3_ AsO_3_ (pK_a1_ = 9.22) and cannot interact with the positively charged methylimidazolium groups of the sorbent. In the pH range of 3–8, the retention of As(V), DMAs, and MMAs ions increased at higher pH values, due to the increasing fraction of charged As forms (As(V): H_2_ AsO_4_^−^, HAsO_4_^2−^, AsO_4_^3−^ (pKa_1_ = 2.22, K_a2_ = 6.97, pK_a3_ = 11.5); DMAs: (CH_3_)_2_ AsO_2_^−^ (pK_a_ = 6.3); MMAs: CH_3_ AsO_3_ H^−^, CH_3_ AsO_3_^2−^ (pK_a1_ = 4.1, pK_a2_ = 9.02)). The quantitative sorption of As(V), DMAs, and MMAs ions on the poly(MIA) was achieved at pH 8. After pH 8, the degree of sorption of As(V), DMAs, and MMAs decreased even though they were negatively charged, which is probably related to the strong competition between OH^−^ and the anions of the studied arsenic species for the binding sites. Finally, a pH of 8 was selected as optimal for the quantitative sorption of As(V), DMAs, and MMAs on the poly(MIA). The experiments performed for the kinetics of sorption showed that the quantitative retention of As(V), DMAs, and MMAs was achieved for about 20 min.

In the second step, the desorption behavior of the retained As(V), DMAs, and MMAs from the polymer gel particles was investigated using various concentrations of HCl and CH_3_COOH solutions as desorption reagents. The results obtained are summarized in Table 1. As seen, all studied arsenic species (As(V), DMAs, and MMAs) were completely eluted with 2 mol/L HCl from the poly(MIA). However, the mild elution reagent, 1 mol/L acetic acid, successfully eluted only the organic As species (DMAs and MMAs), while under these conditions, less than 3% of the As(V) adsorbed on the poly(MIA) was desorbed from the sorbent. Thus, a two-step elution of the retained arsenic species could be suggested for the selective separation of inorganic As(V) from organic As species—1 mol/L CH_3_ COOH for the elution of MMAs and DMAs, while inorganic As(V) remained on the sorbent, and the next elution step including 2 mol/L HCl for As(V). 

The kinetics of the desorption processes of As(V), DMAs, and MMAs was investigated by the procedure described in Section 3.4, with 25 mg of poly(MIA) for a time range of 5 to 60 min. Quantitative desorption (*D*_E_ > 95%) was reached at 20 min. 

The highest sample volume for which the quantitative retention of As(V), DMAs, and MMAs was still found is an important parameter responsible for the calculation of the enrichment factor. Experiments were performed for sample volumes of 20–50 mL. The results obtained show that the degree of sorption for As(V), DMAs, and MMAs was about 95% for the 40 mL sample volume, which dropped down to 80% for the 50 mL sample volume. 

### 2.3. Effect of Initial As(V), DMAs, and MMAs Concentrations and Adsorption Isotherms

**Figure 4:**
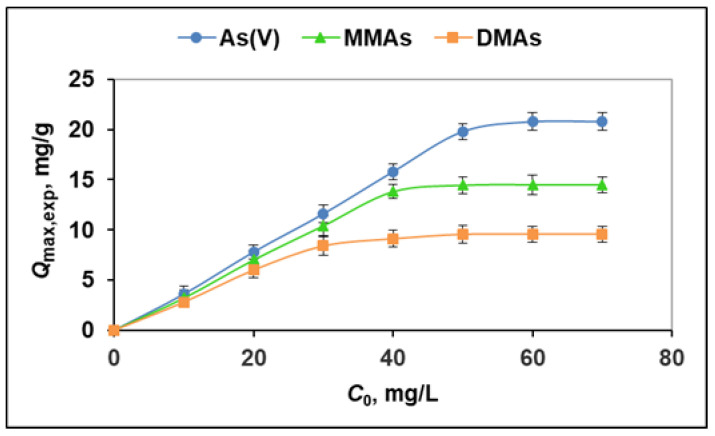
Effect of the initial concentration of As(V), DMAs, and MMAs on the adsorption capacity of poly(MIA) (pH 8; contact time: 20 min; temperature: 25 °C).

In order to evaluate the effect of the initial As(V), DMAs, and MMAs concentration on the adsorption capacity of poly(MIA), batch experiments were conducted according to the procedure described in Section 3.5. The results obtained show that the amount of adsorbed arsenic species per unit mass of the sorbent increased with the their initial concentrations and reached plateau values, determining their maximum experimental adsorption capacity, *Q*_max,exp_ (Figure 4, Table 2). The *Q*_max,exp_ values of the poly(MIA) toward As(V), DMAs, and MMAs were 20.78, 9.58, and 14.50 mg/g, respectively.

Three adsorption isotherm models (Langmuir, Freundlich, and Dubinin–Radushkevich) were tested to describe the relationship between the equilibrium concentration and adsorption capacity during the adsorption process. It is known that the Langmuir isotherm model assumes monolayer adsorption where all binding sites have the same affinity for the adsorbate. The Freundlich model describes multilayer adsorption with a non-uniform distribution of heat of the adsorption and affinities on the heterogeneous surface. The Dubinin–Radushkevich model is applied to express the adsorption mechanism with a Gaussian energy distribution onto a heterogeneous surface adsorption mechanism [31]. The linearized equations of these isotherm models are expressed by Equations (1)–(3) as follows:

(1) $$\mathrm{Langmuir}\mathrm{isotherm}\mathrm{model}:\;\;\frac{C_{e}}{Q_{e}}=\frac{C_{e}}{Q_{\max}}+\frac{1}{b.Q_{\max}},$$

(2) $$\mathrm{Freundlich}\mathrm{isotherm}\mathrm{model}:\;\;\ln Q_{e}=\ln k_{F}+n^{-1}.\ln C_{e},$$

(3) $$\mathrm{Dubinin}–\mathrm{Radushkevich}\;\mathrm{isotherm}:\;\;{\ln Q}_{e}={\ln Q}_{\max\;}-\beta .\varepsilon^{2}\;\;\;\;$$

where *C*_e_ (mg/L) is the equilibrium concentration of the As(V), DMAs, or MMAs in the solution; *Q*_e_ (mg/g) is the adsorption capacity of the poly(MIA) toward As(V), DMAs, or MMAs ions at equilibrium; *Q*_max_ (mg/g) is the calculated maximum adsorption capacity; *b* (L/mg) is the Langmuir constant; *k*_F_ and n are the Freundlich constants incorporating all factors that affect the adsorption process, such as capacity and intensity; *β* is the constant of the sorption energy (mol^2^/kJ^2^), and *ε* (kJ/mol) is the Polanyi potential, which is described as:

(4) $$\varepsilon =RT\;\ln\left[1+\frac{1}{C_{e\;}}\right],$$

where *T* is the temperature of the solution (K), and *R* is the gas constant and equal to 8.314 (J/mol∙K).

The constant *β* gives the mean free energy *E*_DR_ (kJ/mol) of adsorption per molecule of the sorbate when it is transferred to the surface of the solid from infinity in the solution and can be computed using the relationship:

(5) $$E_{DR}=\frac{1}{\sqrt{2\beta }}$$

The obtained results are presented graphically in Figure S2, and the parameters of each model are shown in Table 2. The analysis of the data presented in Table 2 show that the correlation coefficients obtained for the Langmuir isotherms (*R*^2^: 0.9931, 0.9981, and 0.9937 for As(V), DMAs, and MMAs, respectively) have higher values compared to the values obtained when the experimental data were modeled using the Freundlich and Dubinin–Radushkevich isotherm models. This might be accepted as proof that the sorption process occurs as a surface monolayer on homogeneous sites. The calculated adsorption capacities *Q*_max,_ obtained by the Langmuir isotherm model agree very well with the experimentally obtained values, thus confirming the validity of the assumptions for adsorption on monomolecular layers.

To predict the favorability of an adsorption system, the essential characteristics of the Langmuir equation can be expressed in terms of a dimensionless factor, *R*_L_, defined as [31]:

(6) $$R_{L}=\frac{1\;}{1+b.C_{0}},$$

According to the literature, the adsorption isotherm might be irreversible, favorable, linear, or unfavorable if *R*_L_ = 0, 0 < *R*_L_ < 1, *R*_L_ = 1, or *R*_L_ greater than 1, respectively [31]. The results obtained for the *R*_L_ values (Table 2) are in the range of 0 < *R*_L_ < 1, indicating that the adsorption of As(V), DMAs, and MMAs ions on poly(MIA) is favorable.

The Dubinin–Radushkevich isotherm model (Equation (4)) is usually applied to identify adsorption type (chemisorption, physisorption or ion exchange). Information about the nature of the interactions between the sorbent functional groups and the sorbate species can be obtained by evaluating the mean free energy of adsorption, *E*_DR_ (Equation (5)). The value of *E*_DR_ predicts the type of adsorption process as physical (*E*_DR_ < 8 kJ/mol), chemisorption (*E*_DR_ > 16 kJ/mol) or chemical ion exchange (*E*_DR_ = 8–16 kJ/mol) [32]. The data for *E*_DR_ given in Table 2 (2.16, 1.18 and 1.67 kJ/mol) show that adsorption of As(V), D MAs and MMAs ions onto poly(MIA) particles occurs by a physisorption mechanism. However, the chemical structure of poly(MIA (strongly basic anion exchanger)) predicts the ion exchange process to describe the adsorption of As(V), DMAs and MMAs anions on the surface of gel particles. This discrepancy might be explained by taking into account the values of *R*^2^ for the Dubinin–Radushkevich model which are 0.8355, 0.9218 and 0.9003, suggesting that the experimental adsorption data are not fitted to this model and most probably the *E*_DR_ values fall outside the confidence interval. There are number of studies that report a similar discrepancy between the calculated *E*_DR_ values and the predicted sorption mechanism when compared to the real one observed through experimental results [33,34,35]. The important point recommended for consideration in such cases is investigation of the isotherm model and in addition careful study by characteristic techniques of chemical structure and surface properties of the sorbent and analyte binding behavior [36]. It might be concluded that despite the calculated *E*_DR_ values the kinetic data were best fitted by the pseudo-second-order kinetic model (Section 2.4) which confirms the conclusion that the most likely mechanism for adsorption of As(V), DMAs and MMAs on poly(MIA) sorbent is ion exchange. 

### 2.4. Effect of Contact Time and Modeling of the Adsorption Kinetics

**Figure 5:**
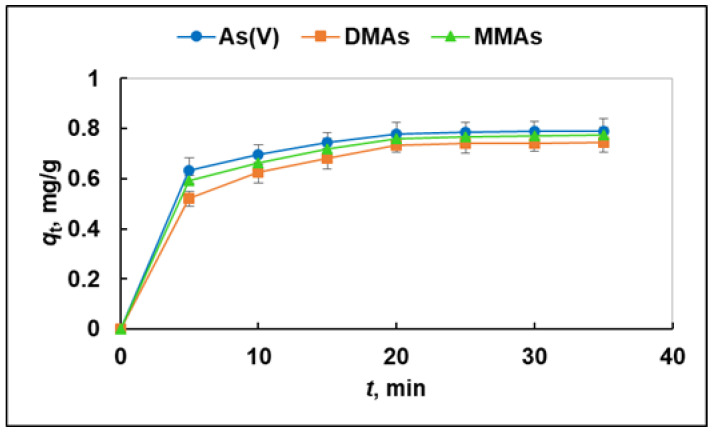
Adsorption kinetics plots of poly(MIA) toward As(V), DMAs, and MMAs (pH 8; sorbent dose = 25 mg/10 mL; *C*_0_ = 2 μg/L, temperature = 25 °C).

The rate of adsorption is one of the important factors for evaluating the sorbent efficiency. In this work, kinetics experiments were carried out for poly(MIA) under the following conditions: 25 mg/10 mL adsorbent dose, 2 μg/mL concentrations of As(V), DMAs, and MMAs ions, pH 8, and temperature 25 °C. The samples were shaken vigorously for durations of 5, 10, 15, 20, 25, 30, and 35 min to determine the effect of the contact time on the sorbent binding capacity. It can be seen that the adsorption capacity of poly(MIA) toward As(V), DMAs, and MMAs increased rapidly in the first 5 min, then increased at a slower pace and remained unchanged after 20 min (Figure 5). The initial fast adsorption was due to the presence of a larger number and more easily accessible specific binding sites on the polymer gel surface. 

In order to determine the controlling mechanisms of the adsorption process, such as mass transfer and chemical reaction, pseudo-first-order and pseudo-second-order kinetic models were applied to fit the data obtained from the adsorption kinetic experiments. The PFO model postulates that the rate of occupation of the adsorption sites is proportional to the number of unoccupied sites, while PSO model is based on the assumption that the adsorption rate is controlled by the chemical adsorption mechanism [37]. The linear forms of the equations for these models can be represented as:

pseudo-first-order model:    ln(*q*_e_ − *q*_t_) = ln*q*_e_ − *k*_1_.*t*, (7)

(8) $$\text{pseudo-second-order}\;\mathrm{model}:\;\;\;\frac{t}{q_{t}}=\frac{1}{k_{1}.q_{e}^{2}}+\frac{t}{q_{e}}$$

where *q*_e_, *q*_t_—amounts of As(V), DMAs and MMAs ions retained per mass unit of sorbent at equilibrium and at time *t*, (mg/g), respectively; *k*_1_, *k*_2_—rate constants of pseudo-first-order kinetics model (1/min) and pseudo-second-order kinetics model (g/mg∙min), respectively.

Figure S3a,b presents the linear plots of the pseudo-first-order and pseudo-second-order models for the sorption of As(V), DMAs, and MMAs ions onto the poly(MIA). Their corresponding kinetic parameters and correlation coefficients are listed in Table 3. A comparison of the results obtained shows that the pseudo-second-order equation appears to be the better-fitting model because it has higher correlation coefficients *R*^2^, and the calculated value of *q*_e,calc_ is closer to the experimental result (*q*_e,exp_). These results prove that the rate-limiting step is the chemisorption of As(V), DMAs, and MMAs ions onto the polymer gel, thus confirming strong interactions of the methylimidazolium fragments in the poly(MIA) with the studied ions.

An evaluation of the involvement of the diffusion process in the adsorption of As(V), DMAs, and MMAs ions on poly(MIA) was performed using the intra-particle diffusion model. This model can be represented by following equation [38]:

(9) $$q_{t}=k_{\mathrm{diff}\;}.\;t^{1/2}+C$$

where *k*_diff_ (mg/g∙min^1/2^)is the intra-particle diffusion rate constant, and intercept *C*, obtained by the extrapolation of the linear portion of the plot of *q*_t_ versus *t*^1/2^, is an indicator expressing the boundary layer thickness.

Two distinct linear parts of the As(V), DMAs, and MMAs adsorption process on poly(MIA) are observed in the plot *q*_t_ versus *t*^1/2^ (Figure S3), which clearly indicates the involvement of more than one step in the adsorption process. The first region could be related to the external mass transfer of the analyte (from the bulk solution to the adsorption surface), while the second region could be explained by the internal diffusion of analyte into the cavities of the polymer gel [39]. The first sorption step was found to proceed with a higher rate because the *k*_diff_ values were higher in the first adsorption stage than in the second stage (Table 3). The boundary layer thickness values (*C*) are different from zero, indicating that the adsorption of As(V), DMAs, and MMAs ions onto the polymer gels was achieved by surface adsorption, which is controlled by the mass transfer resistance in the external liquid film and by pore diffusion [40].

### 2.5. Analytical Application

The aim of the developed procedure for the selective determination of toxic inorganic As(III) and As(V) and organic arsenic MMAs and DMAs was to ensure their quantification avoiding any usage of additional reagents or subtraction. The proposed procedure is based on the combination of two steps. The first step is the selective adsorption of As(V) and organic MMAs and DMAs on poly(MIA) at pH 8, while As(III), which is not sorbed under this pH, is determined in the effluate. The second step consists of two sequential elutions—the first is the selective elution of organic MMAs and DMAs with 1 mol/L acetic acid and their determination, and the second is the desorption of As(V) with 2 mol/L HCl and its determination. The proposed analytical scheme is visualized in Figure S4. Interference studies were performed with model solutions, containing environmentally relevant concentrations of anions major components of surface waters. Results presented in Table S2 showed that only phosphate present real interference at concentrations above 30 mg/L—rare case for surface waters. In addition, the developed analytical strategy was tested on various types of surface (lake, river), ground, and Black sea waters with model mixtures of arsenic species. For all types of water samples, the recoveries achieved varied between 93 and 105%, confirming the absence of interferences from the matrix ions of waters.

Reusability is a key factor in evaluating the performance of poly(MIA). The recoveries achieved after several adsorption/elution steps were compared, and the results lead to the conclusion that poly(MIA) enables at least five loading–elution cycles, indicating good stability and reusability of the sorbent. 

**Analytical figures of merit**. The analytical characteristics of the optimized method for arsenic speciation are summarized in Table 4. In this study, all optimization measurements for the extraction efficiency of poly(MIA) toward arsenic species were carried out using ICP-OES; however, for the applicability of the developed method and its performance characteristics, ICP-MS was used as an instrumental method. An enrichment factor of 20 was calculated as the ratio between the sample volume and the volume of eluate solution. The limit of determination was calculated as ten times that of the standard deviation of the blank solution (five experiments with 40 mL doubly distilled water adjusted to pH 8, stirred with 25 mg sorbent particles, and further treated with eluate solutions) divided by the slope of the calibration graphs. The range of the precision achieved for different As species, presented in Table S1, is based on the three parallel experiments with river, lake, and sea waters. The good accuracy of the developed analytical procedure was demonstrated by the added–found method for water samples spiked with As(III), As(V), MMAs, and DMAs at different concentration levels (see Table S1). In addition, the certified reference material CRM NRC AQUA 1 was analyzed and the results obtained (total As: certified 0.222 ± 0.014 µg/L, determined 0.219 ± 0.012 µg/L; As(III) + As(V): information value 0.124 µg/L, determined 0.128 ± 0.015 µg/L; MMAs + DMAs: information value 0.064 ± 0.014 µg/L, determined 0.061 ± 0.005 µg/L) confirm the validity and versatility of the proposed analytical procedure. 

### 2.6. Comparison with Other Methods

The characteristics of the developed analytical procedure for As speciation in water samples were compared in (Table S3) with those already published in the literature [16,17,18,19,20,21,22,41,42]. As seen poly(MIA) has a higher capacity than the materials cited in Table S2, with the exception of IL-modified magnetic graphene oxide (MGO-IL) [41]. However, this adsorbent is used for the simultaneous separation and removal only of inorganic As species from water. The developed analytical scheme (Figure S4) is characterized by comparable to or better than other reported in the literature values of LODs for As(III), As(V), DMAs and MMAs (Table S3). 

## 3. Materials and Methods

### 3.1. Reagents and Materials

All used reagents and solvents were of analytical reagent-grade. In order to prepare the poly(MIA) sorbent, glycidyl methacrylate (GMA), trimethylolpropane trimethacrylate (TMPTMA), 2,2′-azobisisobutyronitrile (AIBN), 1-methylimidazole (MIA) (Merck, Darmstadt, Germany), and acetonitrile (ACN) (Labscan, Dublin, Ireland) were used.

High-purity deionized water (DW) produced by the Millipore Milli-Q system (Millipore Corp., Milford, MA, USA) was used to prepare the aqueous solutions for the sorption/desorption experiments. The stock standard solutions of As were: 1000 mg/L As(III) (Fluka, Munich, Germany); 1000 mg/L As(V) (Merck, Darmstadt, Germany); 1000 mg/L monomethylarsonate (MMAs), prepared by dissolving disodium methylarsonate hexahydrate (CH_3_ AsO(ONa)_2_·6H_2_O) (Carlo Erba, Milan, Italy); 1000 mg/L dimethylarsinate (DMAs), prepared by dissolving of sodium cacodylate trihydrate ((CH_3_)_2_ As(O)ONa·3H_2_O) (Sigma Aldrich, St. Louis, MO, USA) in DW. The working standard solutions were prepared weekly and kept refrigerated at 4 °C.

AQUA-1, a drinking water certified reference material (CRM) from the National Research Council Canada (NRC) was used.

The pH of the sample solutions was adjusted with nitric acid or sodium hydroxide solution (Merck, Darmstadt, Germany). The elution solutions were prepared with acetic acid and hydrochloric acid (Merck, Darmstadt, Germany).

### 3.2. Apparatus

A scanning electron microscope (SEM, JEOL JSM-5500, Tokyo, Japan) was used to study the morphological characterization of the synthesized polymer gels. The specific surface area, total pore volume, and average pore diameter of the polymer particles was determined by N_2_ adsorption–desorption isotherms at 77 K using the Quantachrome NOVA 1200 apparatus (Quantachrome Ltd., Boynton Beach, FL, USA). An automatic CHNS-O elemental analyzer, the EuroEA 3000 (EuroVector, Redavalle, Italy), was used to determine the carbon, hydrogen, and nitrogen (wt.%) contents in the prepared samples. A microprocessor pH meter (Mettler Toledo; Seven Compact S220-K, Greifensee, Switzerland) was used for all pH measurements. An EBA 20 centrifuge (DJB Labcare Ltd., Newport Pagnell, UK) was used to separate the polymer particles and the solution containing the extracted arsenic species in the batch experiments. The FTIR spectra were recorded on the Nicolet 6700 FT-IR spectrometer, Thermo Scientific (Madison, WI, USA). The samples were analyzed as KBr pellets.

The concentrations of the As species in the model experiments investigating the extraction efficiency of the poly(MIA) sorbent, the optimization of the pH values, and the choice of eluent were measured by an inductively coupled plasma optical emission spectrometer (ICP-OES, Jobin Yvon Ultima 2). All measurements were carried out with at least three replicates.

### 3.3. Synthesis of Poly(MIA)

The poly(MIA) particles were prepared as described earlier using a two-step synthesis procedure with some modifications [43]. Initially, GMA (0.546 mmol), TMPTMA (0.894 mmol), and AIBN (initiator, 32 mg) were dissolved in acetonitrile (porogenic solvent, 25 mL). The prepared solution was saturated with dry nitrogen for 15 min, and precipitation copolymerization was carried out at temperature of 60 °C for 24 h. The polymer gel particles obtained, poly(GMA), were recovered by centrifugation and washed with acetonitrile to remove unreacted monomers and other ingredients. Finally, the poly(GMA) particles were dried in a vacuum oven at 60 °C.

In the next step, functionalization of the poly(GMA) particles with MIA was performed in the following way: MIA (6.3 mmol) and NaOH (0.1 g) were dissolved in a mixture of ethanol (20 mL) and H_2_O (25 mL) in a flask, and the poly(GMA) particles (0.5 g) were added. The suspension was stirred and refluxed at 80 °C for 12 h. The obtained product (called poly(MIA)) was recovered by centrifugation and washed with ethanol to remove the unreacted MIA. Finally, the prepared material was dried at 60 °C for 8 h under vacuum. The synthesis of poly(MIA) is presented schematically in Figure 1.

### 3.4. Optimization of SPE Conditions

The optimal SPE conditions (solution pH, concentration of the eluents, sorption, and elution time) ensuring the maximum extraction efficiency were evaluated by a batch procedure, as follows: A portion of the standard solution containing 20 μg As(III), As(V), DMAs, or MMAs was added to a 10 mL test solution and adjusted to a desired pH value with NaOH or HNO_3_. About 25 mg of poly(MIA) particles were added to this solution and stirred with an electric shaker for 25 min. The suspension was centrifuged at 5000 rpm, the supernatant removed, and the polymer gel particles were washed twice with DW. The retained arsenic species were eluted from the polymer particles with 5 mL 2 mol/L HCl (for As(V)) and 1 mol/L CH_3_COOH (for DMAs + MMAs). The arsenic content in the effluate (supernatant after sorption) and eluate solutions was measured by ICP-OES.

The degree of sorption (*D*_S_ %) of the As species ions is defined as:

(10) $$D_{S}=\frac{A_{i}\;–A_{\mathrm{eff}}}{A_{i}}\times 100$$

where *A*_eff_ (μg) is the As amount in the effluate solution after extraction by poly(MIA) from a solution with an initial As amount *A*_i_ (μg).

The degree of elution (*D*_E_, %) of the adsorbed As species ions was calculated using the following equation:

(11) $$D_{E}=\frac{A_{\mathrm{el}}\;}{A_{i}–A_{\mathrm{eff}}}\times 100,$$

where *A*_el_ (μg) is the amount of As in the solution after the elution process.

### 3.5. Isotherm and Kinetic Studies

The adsorption capacity of the obtained poly(MIA) was found by the following procedure: 25 mg of the adsorbent was mixed with 10 mL of As(V), DMAs, or MMAs solution with an increasing initial concentration (20, 30, 40, 50, 60, 70, and 80 μg/mL) under optimal conditions at a temperature of 25 °C. The equilibrium As(V) concentration after adsorption was measured by ICP-OES. The maximum adsorption capacity of the poly(MIA) (*Q*_max_,_exp_) is defined as the amount of the adsorbed As(V), DMAs, or MMAs ions per gram of the polymer gel and can be calculated by the following equation:

(12) $$Q_{\max,\exp}=\frac{(C_{0}–C_{e}).V\;}{m},$$

where *Q*_max,exp_ (mg/g) is the mass of As(V), DMAs, or MMAs ions adsorbed per unit mass of the sorbent; *V* (L) is the volume of the solution; *m* (g) is the mass of the sorbent; *C*_0_ and *C*_e_ (mg/L) are the initial and equilibrium concentrations of the As(V), DMAs, or MMAs ions in the solution.

The kinetics of the As(V), DMAs, and MMAs sorption was investigated in a batch system. First, 10 mL of aqueous solution, containing 20 μg As(V), DMAs, or MMAs, was treated with 25 mg of polymer gel particles at pH 8 and temperature of 25 °C for 5–35 min. Then, the sample was vigorously shaken, and 20 μL aliquots of the supernatant solution were taken at 5 min intervals. The arsenic concentration in these experiments was measured by ICP-OES.

### 3.6. Analytical Procedure

A volume of 40 mL of the water sample was mixed with 25 mg poly(MIA) and stirred for 20 min. The solution was centrifuged for 2 min at 500 rpm, and the As(III) was determined in the supernatant. The remaining sorbent particles were washed with doubly distilled water and stirred with 2 mL 1 mol/L acetic acid for 2 min. After centrifugation for 2 min, the supernatant was removed for the measurement of the sum of MMAs + DMAs. The sorbent particles were washed with distilled water and stirred with 2 mL 2 mol/L HCl. After centrifugation, the As(V) was measured in the supernatant. Arsenic concentration in these experiments was measured by ICP-MS.

## 4. Conclusions

A non-chromatographic analytical procedure was developed for arsenic speciation in different types of surface waters. The separation of As(III), As(V), MMAs, and DMAs was achieved by the selective sorption/elution on/from the surface of newly synthesized ionic liquid modified polymeric gel (poly(MIA)). The sorbent composition and structure were characterized by elemental analysis, FTIR, SEM, and nitrogen adsorption–desorption measurements. Experimental results and the calculated adsorption capacities *Q*_max_ revealed the adsorption of As(V), DMAs, and MMAs ions on homogeneous sites on the surface of the sorbent in a monomolecular layer. Kinetic studies proved that the rate-limiting step was the chemisorption (ion exchange) of As(V), DMAs, and MMAs ions onto the polymer gel surface, thus confirming strong interactions of the methylimidazolium fragments in poly(MIA) with these ions.

The ionic liquid modified polymeric gel was used as a solid phase for arsenic speciation in water samples. The advantages of the proposed analytical procedure are: (i) no need to use additional chelate complex forming reagent (ii) no need to use reagents for pre-oxidation or pre-reduction of the arsenic species; (iii) all analytical steps might be performed in one analytical vessel (centrifugation tube); (iv) determination limits achieved ensured successful application for arsenic content assessment in monitoring campaign.

The developed analytical method was validated by the analysis of certified reference material in this way confirming the accuracy of the results obtained. 

## Figures and Tables

**Table 1:** Degree of elution (%) for As(V), DMAs, and MMAs from poly(MIA) using different eluents (5 mL).

Eluent Solution	*D*_E_, %		
	As(V)	DMAs	MMAs
0.5 mol/L CH_3_COOH	<3	86 ± 3	87 ± 2
1.0 mol/L CH_3_COOH	<3	99 ± 2	99 ± 2
0.5 mol/L HCl	65 ± 3	99 ± 2	98 ± 2
1.0 mol/L HCl	77 ± 3	99 ± 2	99 ± 2
2.0 mol/L HCl	99 ± 2	99 ± 2	99 ± 2
3.0 mol/L HCl	99 ± 2	99 ± 2	99 ± 2

**Table 2:** Experimental and fitting parameters of the various isotherm models for the adsorption of As(V), DMAs, and MMAs ions onto the poly(MIA) at temperature 25 °C.

Adsorption Isotherm Model	Parameters	As(V)	DMAs	MMAs
Experimental adsorption capacity	*Q*_max,exp_ (mg/g)	20.78	9.58	14.50
Langmuir	*Q*_max_ (mg/g)	20.53	9.64	14.45
	*b* (L/mg)	0.45	0.76	0.69
	*R* ^2^	0.9931	0.9981	0.9937
	*R*_L_	0.03–0.10	0.02–0.12	0.02–0.13
Freundlich	*k*_F_	6.78	8.02	6.37
	*n*	2.74	9.92	4.00
	*R* ^2^	0.9755	0.9505	0.9086
Dubinin–Radushkevich	*Q*_max_ (mg/g)	14.65	8.76	12.12
	*β* (mol^2^/kJ^2^)	0.11	0.36	0.18
	*E*_DR_ (kJ/mol)	2.16	1.18	1.67
	*R* ^2^	0.8355	0.9218	0.9003

**Table 3:** Parameters of pseudo-first-order, pseudo-second-order, and intra-particle diffusion models for adsorption of As(V), DMAs, and MMAs ions onto the poly(MIA). (pH 8; sorbent dose = 25 mg/10 mL; *C*_0_ = 2 μg/L, temperature = 25 °C).

Model	Parameters	As(V)	DMAs	MMAs
Experimental adsorption capacity	*q*_e,exp_ (mg/g)	0.7936	0.7640	0.7816
Pseudo-first-order model	*q*_e,calc_ (mg/g)	0.2988	0.3175	0.3103
	*k*_1_ (1/min)	0.1292	0.0882	0.1135
	*R* ^2^	0.9647	0.9320	0.9629
Pseudo-second-order model	*q*_e,calc_ (mg/g)	0.8317	0.8095	0.8244
	*k*_2_ (g/mg∙min)	0.7222	0.4479	0.5860
	*R* ^2^	0.9936	0.9994	0.9995
Intra-particle diffusion model Region 1 (from 5 to 20 min)	*k*_dif*f*_ (mg/g∙min^1/2^)	0.0650	0.0895	0.0757
	*C* (mg/g)	0.4886	0.3285	0.4240
	*R* ^2^	0.9973	0.9854	0.9990
Intra-particle diffusion model Region 2 (from 20 to 35 min)	*k*_diff_ (mg/g∙min^1/2^)	0.0035	0.0086	0.0154
	*C* (mg/g)	0.7683	0.7395	0.6556
	*R* ^2^	0.9355	0.8304	0.7701

**Table 4:** Analytical characteristics of the proposed method.

Determination Limit, ng/L			Relative Standard Deviation, % Concentration Range 0.01–20 µg/L		
As(V)	As(III)	(DMAs + MMAs)	As(V)	As(III)	(DMAs + MMAs)
1	10	1	4–10	5–8	5–10

## Data Availability

Data are contained within the article and Supplementary Materials.

## Supplementary Materials

The following supporting information can be downloaded at: https://www.mdpi.com/article/10.3390/molecules29040898/s1, Figure S1: FTIR spectra of poly(GMA) and poly(MIA); Figure S2: Langmuir (a), Freundlich (b), and Dubinin–Radushkevich (c) isotherms for adsorption of As(V), DMAs, and MMAs on the poly(MIA); Figure S3: Adsorption kinetics of As(V), DMAs, and MMAs ions onto the poly(MIA) at concentration of 2 mg/L, pH 8, temperature of 25 °C, and adsorbent dose of 25 mg: (a) pseudo-first-order model; (b) pseudo-second-order model; (c) intra-particle diffusion model; Figure S4: Scheme of analytical procedure for As(III), As(V), DMAs, and MMAs separation and determination in water samples using poly(MIA); Table S1: Added–found method applied for water samples (three parallel determinations). Table S2 Recoveries achieved for arsenic species (model solutions) in the presence of environmentally relevant concentrations of anions in surface waters (three parallel experiments); Table S3: Comparison of SPE methods using different sorbent materials for arsenic speciation.
