# Supplementary material for: Ionic Liquid Modified Polymer Gel for Arsenic Speciation
Source: Molecules. 2024 Feb 18;29(4):898. doi: 10.3390/molecules29040898 (PMC10892277; doi:10.3390/molecules29040898)
Supplement: Supplementary file 1 [file molecules-29-00898-s001.zip › molecules-2815320-supplementary.pdf]

Supplementary Materials

Ionic liquid modified polymer gel for arsenic speciation

Ivanka Dakova * and Irina Karadjova

Faculty of Chemistry and Pharmacy, University of Sofia "St. Kliment Ohridski", 1, J. Bourchier Blvd., 1164 Sofia, Bulgaria
* Correspondence: i.dakova@chem.uni-sofia.bg (I.D.);

Figures

Section: *2.1. Synthesis and characterization of poly(MIA)*

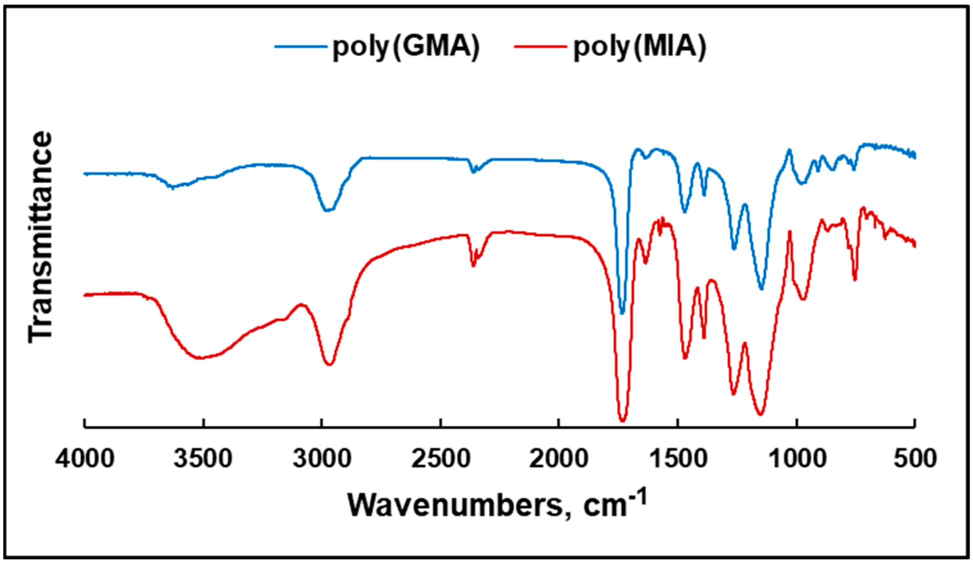

Figure S1. FTIR spectra of poly(GMA) and poly(MIA)**.**

Section S2.4.1. Adsorption isotherm models

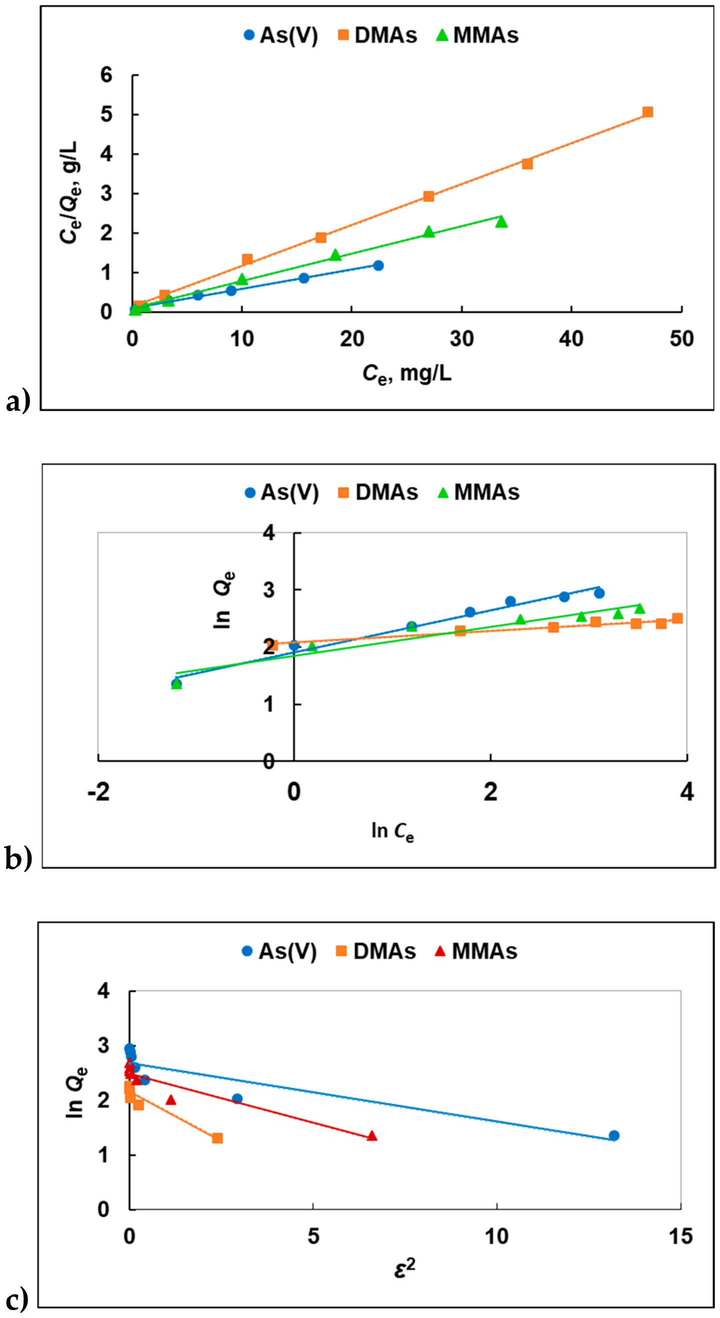

Figure S2. Langmuir (a), Freundlich (b) and Dubinin-Radushkevich (c) isotherms for adsorption of As(V), DMAs and MMAs on the poly(MIA).

Section S2.4.2. Modelling of As(V), DMAs and MMAs sorption kinetics

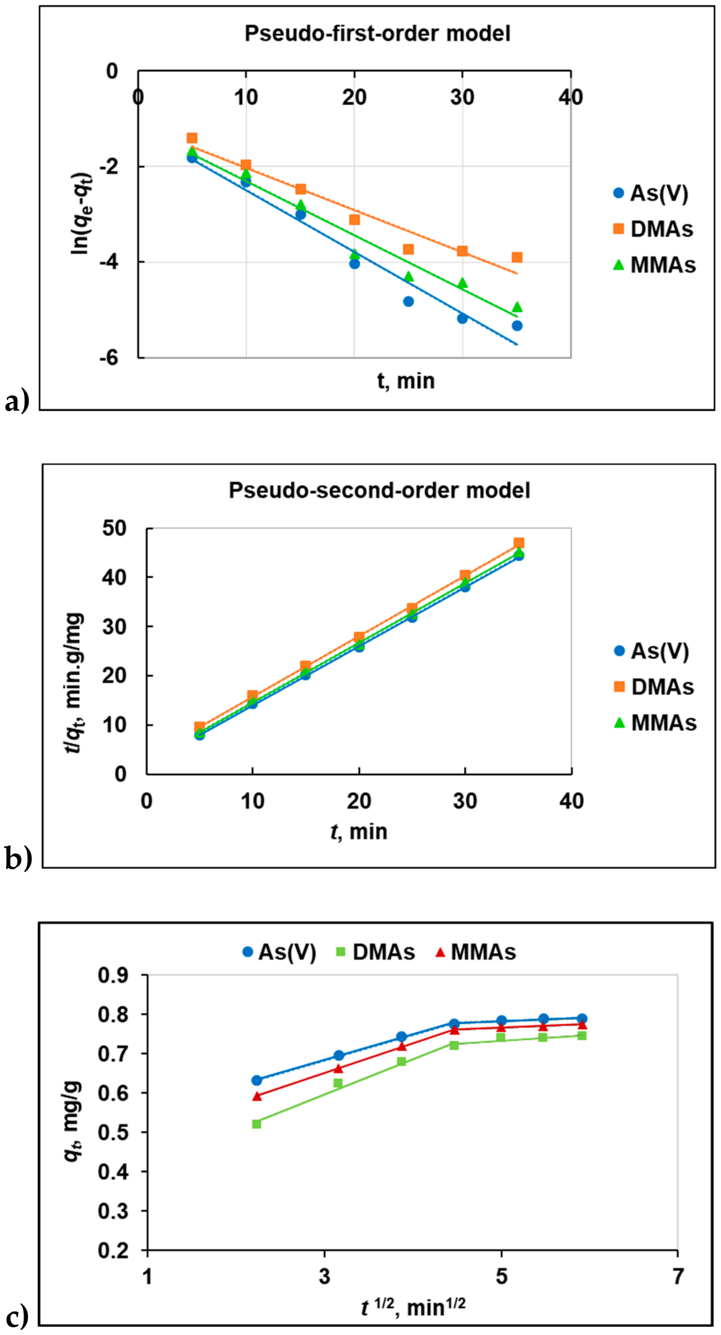

a)
b)
c)

Figure S3. Adsorption kinetics of As(V), DMAs and MMAs ions onto the poly(MIA) at concentration 2 mg/L, pH 8, temperature 25 °C, and adsorbent dose 25 mg: (a) pseudo-first order; (b) pseudo-second order model; (c) intra-particle diffusion model.

Section: *2.5. Analytical Application*

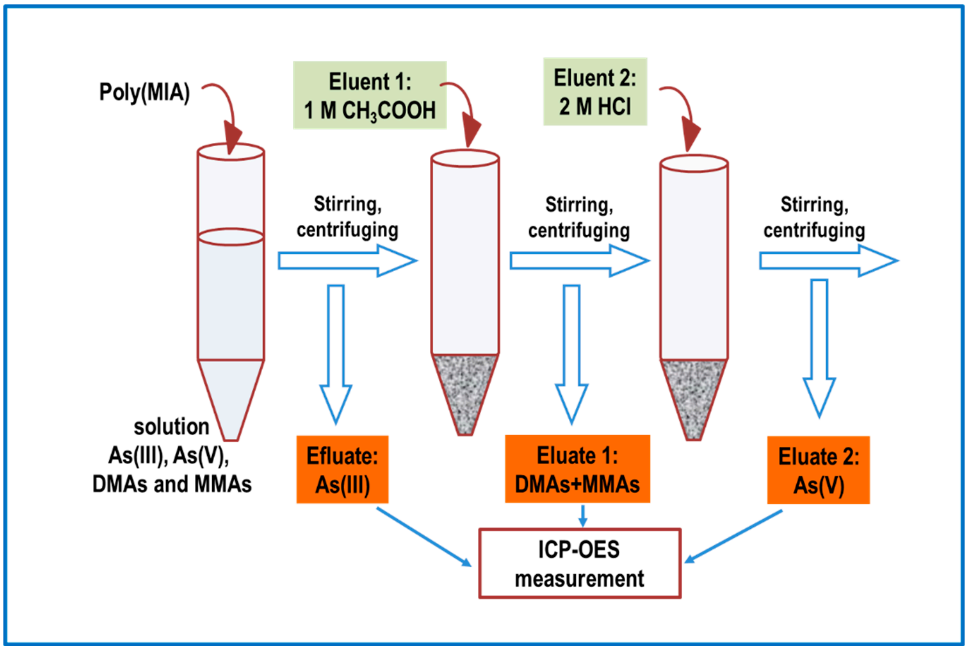

Figure S4. Scheme of the analytical procedure for As(III), As(V), DMAs and MMAs separation and determination in water samples using poly(MIA).

Tables

Section: *2.5. Analytical Application*

Table S1. Added/Found method applied for water samples (three parallel determinations)

Sample	Added, μg/L			Found, μg/L		
	As(V)	As(III)	(DMAs+MMAs)	As(V)	As(III)	(DMAs+MMAs)
Lake Ognyanovo	1.0	-	0.1	1.1±0.1	<DL	0.09±0.01
	5.0	1.0	0.2	4.9±0.2	1.2±0.1	0.18±0.02
	0.5	0.2	0.1	0.4±0.1	0.18±0.02	0.11±0.02
River Iskar	1.0	-	0.1	0.9±0.1	<DL	0.12±0.02
	5.0	1.0	0.2	4.8±0.2	1.1±0.1	0.19±0.02
	0.5	0.2	0.1	0.5±0.1	0.21±0.02	0.09±0.02
Black sea water	2.0	0.1	0.05	1.9±0.2	0.11±0.01	0.044±0.003
	4.0	0.2	0.05	3.9±0.3	0.19±0.02	0.052±0.003
	10.0	0.5	0.1	10.1±0.9	0.51±0.03	0.09±0.01

Table S2. Recoveries achieved for arsenic species (model solutions) in the presence of environmentally relevant concentrations of anions in surface waters (three parallel experiments).

**Interferent**	**As(V)**	**MMA**	**DMA**
Cl^- (500 mg/L)	>99	>99	>99
NO_3^- (50 mg/L)	98±2	96±2	97±2
HCO_3^- (300 mg/L)	96±4	94±3	95±3
SO_4^{2-} (200 mg/L)	92±4	93±4	95±4
PO_4^{3-} (20 mg/L)	93±5	92±4	93±4

Section: *2.6. Comparison with other methods*

Table S3. Comparison of SPE methods using different sorbent materials for arsenic speciation.

Sorbent material	Separated species	pH	Sorbent mass (mg) / adsorbate concentration (mg/L)	Sorption capacity, mg/g	Analytical technique	Sample	LOD (µg/L)	Ref.
Alltech SAX and SCX columns	As(III)	6.5	500 / -	-	NAA	waters	0.9	[16]
	As(V)						1.7	
	DMAs						3.8	
	MMAs						1.6	
Three types immobilized macrocyclic materials	As(III)	7	- / 100 [1]		GF-AAS	waters	0.06	[17]
	As(V)			0.31-0.40 [2]			0.06	
	DMAs						0.05	
	MMAs						0.05	
Three types of resins: SBAE, HY-Fe and HY-AgCl	As(III)	7 – 9	1000 / 0.5	4.15	ICP-MS	waters	0.2	[18]
	As(V)			0.37			0.2	
	DMAs			0.10			0.2	
	MMAs			0.144			0.2	
A dual column packed with a magnetic MFC and MFC-SH	As(III)	6	- / 10 [4]	4.2	ICP-MS	Waters, hair, urine, SCC-7 cells	7.1 [5]	[19]
	As(V)			3.5			4.8 [5]	
	DMAs			2.4			6.3 [5]	
	MMAs			3.1			3.8 [5]	
tri-n-butylphosphine modified polymer microsphere	As(III)	7	- /0.1 [4]	-	HPLC–ICP-MS	waters	1.2 [5]	[20]
	As(V)						0.91 [5]	
	DMAs						0.96 [5]	
	MMAs						0.82 [5]	
MGO-PSTH	As(III)	1	- / 5 [4]	1.6	HPLC-ICP-MS	Seawater	1.1 [5]	[21]

	As(V)			5.0			0.2 [5]	
	AsBet			1.1			3.8 [5]	
	DMAs			3.2			0.5 [5]	
NH_2-UVM	As(III)	3.5	10 / 0.1[4]	39.3 [3]	ETAAS	Waters,		[22]
	As(V)			123.2		human	3.3 [5]	
	TOA			44.2 [3]		urine		
MGO-IL	As(Ⅲ)	9	20 / 50	160.65	AFS	water	-	[41]
	As(V)	2		104.13				
As(V) ion surface imprinted polymer	As(V)	3	20 / 10–800	39.5	UV-vis	waters	0.26	[42]
ionic liquid modified polymer gel	As(III)	8	25 / 2		ICP-MS	Waters	10 [5]	This work
	As(V)			20.78			1[5]	
	DMAs			9.58			1[5,6]	
	MMAs			14.50				

[1] μM; [2] mmol/g; [3] μg/g; [4] μg/L; [5] ng/L; [6] DMAs+ MMAs.

Abreviations: AsBet - arsenobetaine; HY - hybrid resin; MFC - magnetic metal-organic framework composite; MFC-SH - mercapto-functionalized MFC nanoparticles; MGO-IL 1-butyl-3-methylimidazolium hexafluorophosphate ([BMIM] [PF_6])-modified magnetic graphene oxide; MGO-PSTH - magnetic nanomaterial functionalized with [1,5-bis (2-pyridyl) 3-sulfophenylmethylene] thiocarbonohydrazide; NH_2-UVM - amine-functionalized bimodal mesoporous silica nanoparticles; SAX - strongly anion exchanger; SCX - strongly cation exchanger; SBAE - strong base anion exchange resin; TOA - total organic arsenic (TOA (MMA, DMA, TMAO, AC and AB)
